# Improving Tractor Safety: A Comparison between the Usability of a Conventional and Enhanced Rear-Mounted Foldable ROPS (FROPS)

**DOI:** 10.3390/ijerph191610195

**Published:** 2022-08-17

**Authors:** Lucia Vigoroso, Federica Caffaro, Margherita Micheletti Cremasco, Eugenio Cavallo

**Affiliations:** 1 Institute of Sciences and Technologies for Sustainable Energy and Mobility (STEMS), National Research Council of Italy (CNR), Strada delle Cacce, 73, 10135 Torino, Italy; 2 Department of Education, University of Roma Tre, Via del Castro Pretorio 20, 00185 Rome, Italy; 3 Department of Life Sciences and Systems Biology, University of Turin, Via Accademia Albertina, 13, 10123 Torino, Italy

**Keywords:** agriculture, rollover protective structure, human–machine interaction, occupational safety, usability evaluation

## Abstract

Tractor rollover is the main cause of both fatal and non-fatal injuries in agriculture. The foldable rollover protective structure (FROPS) can help to prevent these injuries. However, in many cases, the FROPS is left in a folded-down position. Human factor and rear-mounted FROPS technical characteristics influence operators’ behavior in roll-bar handling. To improve the FROPS’s comfortable use, the prototype of an enhanced handling system was developed, and its usability was tested and compared with a conventional FROPS. Usability was assessed with 93 novice tractor users through an ad hoc questionnaire (investigating perceived effort, time demand, the posture adopted and satisfaction) and observations (investigating effectiveness and efficiency) during lowering and raising tasks. For both tasks, the participants perceived significantly less effort, less physical discomfort, a higher level of satisfaction and less time demand while operating the enhanced FROPS. Observations showed that the critical issues that emerged for the conventional FROPS were eliminated by adopting the developed and implemented handling system. The developed handling system showed itself to be usable and effective in making the FROPS easier and safer to be operated.

## 1. Introduction

**Figure 1:**
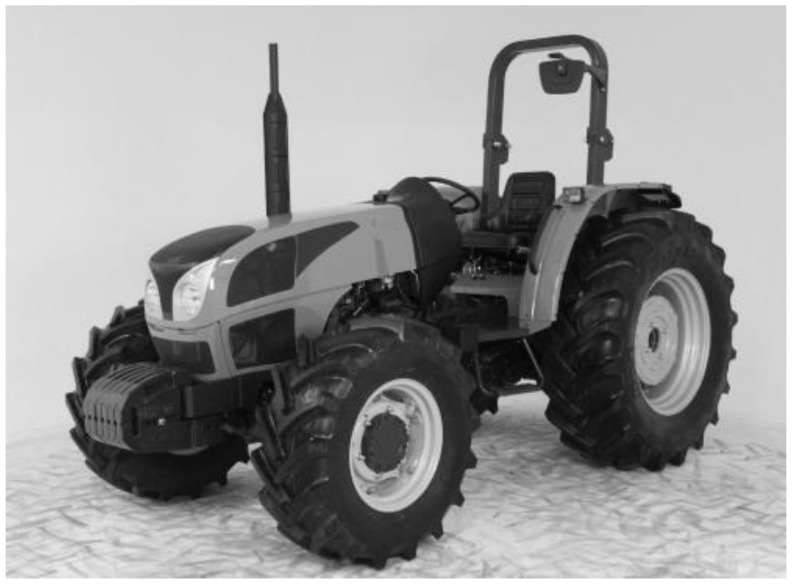
Example of a rear FROPS fitted on a standard tractor (track width > 1150 mm) in the upright position.

The rollover protective structure (ROPS) (i.e., safety cab, frame or roll-bar) is a mechanical structure fitted on agricultural tractors which absorbs the tractor’s impact energy with the ground in case of overturning [1]. The ROPS, together with the seatbelt, has been developed to avoid or limit the risks to the driver resulting from a tractor rollover and the continuous roll of the tractor down a slope, keeping the driver within the clearance zone (i.e., a standardized volume representing the space occupied by the driver when seated inside the envelope of the structure or within a space that is capable of protecting the operator if the tractor overturns) [2,3,4]. When the foldable roll-bar (FROPS) version was introduced on the market, it became largely used in narrow-track tractors (tractors with a track width of no more than 1150 mm [5]) to facilitate tractor operation in low overhead clearance conditions such as low trees (vineyards and orchards) and greenhouses and buildings such as poultry farms, and it is frequently used in standard agricultural tractors (tractors with a track width of more than 1150 mm) to improve the tractors’ mobility, transport and storage and to reduce purchase costs compared with a tractor equipped with a cab [6,7,8] (Figure 1).

Unfortunately, a high rate of injuries and fatalities still occurs during tractor rollover [6,7,9,10,11] when the FROPS is in the folded-down position. Tractor safety education programs insist on the role of the FROPS to prevent injuries, and international standards such as the OECD Standard Codes provide requirements to be fulfilled in the design and testing of front- [3] and rear-mounted [4] FROPSs fitted on narrow-track tractors [5] in terms of accessible zones (i.e., the volume where a standing operator can apply a force to raise or lower the ROPS ([3,4], p. 28)) and allowed forces to guarantee safe and comfortable operation. Despite this, there is a widespread farmers’ propensity to leave the FROPS in the folded-down position [12,13,14], thus compromising the FROPS’s protective value [6]. Previous studies [12,13,14,15] linked this unsafe behavior to the farmers’ view of FROPS handling as a time-consuming, strenuous and uncomfortable operation. This evidence is particularly relevant considering that a FROPS needs to be manually operated. For some authors, new design solutions may convince farmers to change their attitudes toward FROPS adoption [16].

Most of the research on FROPS design is based on technical and engineering aspects to improve the design of the protective structure itself [17,18], focusing on enhancing the strength test results, improving the parameters affecting the tractor’s stability [7,19,20,21] and reducing the actuation forces required to raise and lower the FROPS [16]. For instance, Ayers et al. [22] proposed and tested a FROPS lift-assist mechanism on three tractors with different masses. Even though the study reported that the engineering standards were met, a fatigue test with operators to evaluate the components’ reliability during the typical operation was not performed.

Although the ergonomic issues during FROPS handling have received increasing attention, studies focusing on the human perspective on rear-mounted FROPS operation are still few or often hard to acquire. In Caffaro et al. [14] and Micheletti Cremasco et al. [23], the roles played by the operators’ anthropometric characteristics and the FROPS’s technical features in determining the roll-bar handling were explored. These two studies investigated the critical behaviors and awkward gestures and postures taken by farmers when raising a FROPS mounted on different models of tractors. These studies reported that some parts of the tractors (i.e., the lower links of the rear three-point linkage and the power take-off protection) were used by the operators as a stand to reach and operate the FROPS and that the operators adopted asymmetrical postures while carrying out the operation. In addition, Vigoroso et al. [24] pointed out that some farmers used an additional element fitted on the FROPS, such as the mirror shaft, to overcome the reachability issue and to facilitate FROPS raising and lowering operations. Previous studies [25,26] pointed out that when engineers design a product, they often adopt different perspectives from those of the final users for whom they design it, and their motivation does not usually come from a specific personal problem to be solved. Moreover, as reported by Roto et al. [27], the quality of a product depends mainly on those who experience it.

Regarding agricultural machinery, to fully integrate the users’ needs into the quality system, previous studies suggested that human factors should be considered during the tractor design phase, recommending the adoption of the user-centered design (UCD) approach [28,29]. The UCD approach is an iterative process based on the involvement of users from the early design phases, conceptualizing activities and ideas [30] and taking into account the users’ needs and requirements with the purpose of producing alternative solutions that are highly usable, accessible and positively evaluated by the users [31]. In its broader definition, usability can be defined as the “extent to which a system, product or service can be used by specified users to achieve specified goals with effectiveness, efficiency and satisfaction in a specified context of use” [32]. Thus far, the UCD approach has mainly been applied to improve digital interfaces and enhance assistance systems in highly technological machinery [33,34] and to design more efficient and comfortable working equipment and tools [35,36]. Based on this evidence, usability evaluation can be useful to assess the ease of operation (i.e., effectiveness), functionality, performance (i.e., efficiency) and satisfaction in use [30] of an industrial design product such as the FROPS.

The present study is part of a wider research project in which a UCD approach [30] was adopted to improve the handling of a rear-mounted FROPS fitted on standard (having a minimum wheel track width of 1150 mm or greater) [5] wheeled tractors. A previous paper [14] dealt with the first (plan the human-centered process), second (understand and specify the context of use) and third (specify users’ requirements) phases of the UCD approach, whereas in this paper, the fourth (produce design solutions, namely the prototype) and fifth (design evaluation based on the users’ perspective) phases are described. Thus, the present study reports on the development and implementation of the prototype of a new system to facilitate the raising and lowering operations of a rear-mounted FROPS fitted on units other than narrow-track tractors, where the dimensions of the tractor make such operations hard to be carried out manually from the ground. Then, the usability of a FROPS enhanced with the newly designed system was tested and compared with a conventional FROPS (i.e., without any added components) to highlight any improvement in handling safety and comfort in use.

## 2. Materials and Methods

### 2.1. Participants

Participants were recruited from the Department of Agriculture at the University of Torino (Italy), where 93 male tractor users agreed to be involved in the study. The participants had a mean age of 20.45 years (SD = 3.45, range: 18–38 years), a mean stature of 178.79 cm (SD = 8.96, range: 153–200 cm) and a mean body mass of 73.33 kg (SD = 13.87, range: 55–104 kg). The participants reported a mean farming experience of 4.69 years (SD = 2.02) and operating a tractor equipped with FROPS a few times a year. Participation was voluntary, and no incentives were offered. The Institutional Review Board of the Institute for Agricultural and Earthmoving Machines (which later became the Institute of Science and Technologies for Sustainable Energy and Mobility, approval date: 10 April 2019). The participants gave their written informed consent before their inclusion in the study.

### 2.2. The FROPS Tested in the Study

**Figure 2:**
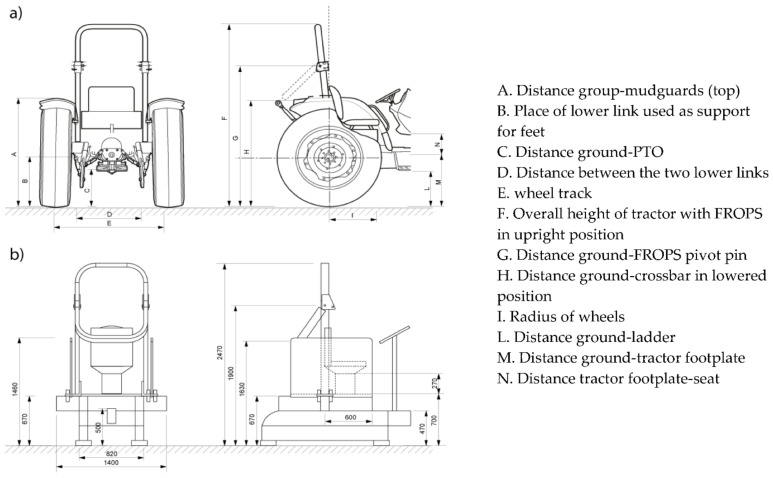
(**a**) Definitions of the dimensions observed on real tractors and (**b**) the dimensions (mm) of the mock-up tractors resulted and built for the study.

In the present study, two structures which simulated standard tractor driving station volumes were designed and built using the average dimensions recorded on tractors in previous in-field investigations [14,23] (Figure 2). The two mock-ups refer to standard tractors [5] with a rear-mounted FROPS, and their design considered the overall height and width of the tractor as well as the dimensions of other components such as the mudguard on the back wheels of the tractor and the steering wheel on the front of the tractor, which could influence the operators’ placement and behavior while performing the targeted tasks. The rear wheels’ clearances, an additional obstacle that can affect the position of the operator, were marked on the floor using white adhesive tape. and the participants were asked not to cross such lines, indicating the spatial limits to comply with during the tasks they had to perform. Micheletti Cremasco et al. [23] in their in-field study observed that the operators used the rear lower links of the rear three-point linkage [37] and the rear power take-off protection [38] as a support for their feet during FROPS handling. Therefore, to ensure that the participants would perform the targeted tasks safely, such components were built with an adequate standing surface able to accommodate at least one foot [4].

One of the two mock-up tractor structures was equipped with a conventional FROPS, whereas the other one had a FROPS fitted with the prototype of a handling system. The handling system to facilitate the FROPS’s raising and folding was designed to comply with the following requirements: (1) none of its parts should enter or infringe upon the clearance zone (the volume defined by the OECD codes and corresponding to the volume of the operator when seated on the tractor in driving position [3,4]), and (2) it should be easy to implement on an existing FROPS.

**Figure 3:**
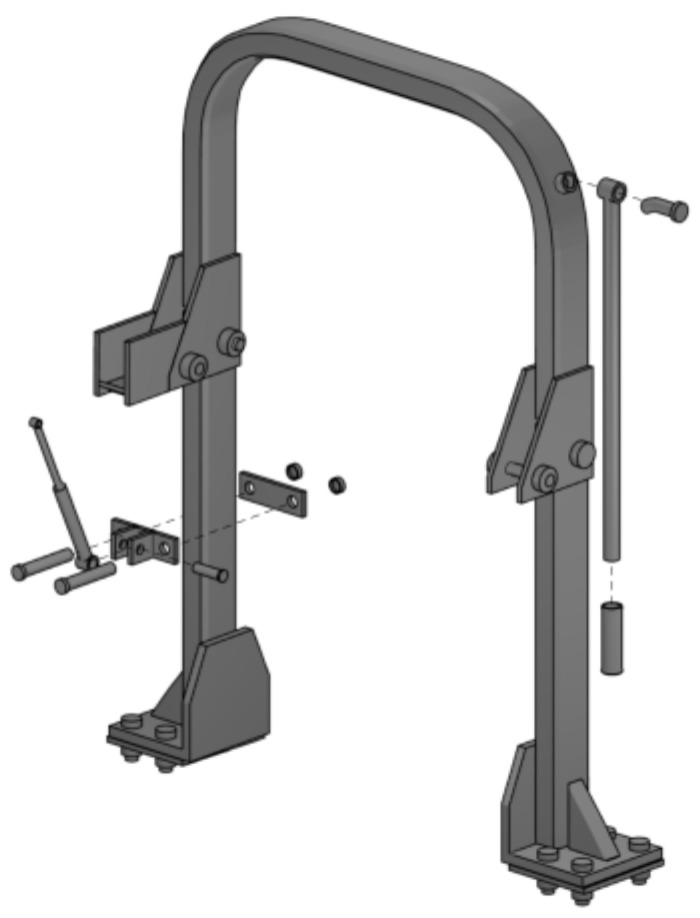
The rod (right) and the gas spring (left) prototyped and mounted on the enhanced FROPS to improve the handling of the upper inverted U-shaped folding steel tube.

The prototyped handling system consisted of a rod and a gas spring fitted on the FROPS. The rod was inserted on a threaded pin fastened on the FROPS and held in place by a nut. This connection allowed free rotation of the rod on the vertical plane, whereas when the rod was in the resting position, it was locked by a retainer placed on the lowest outer part of the FROPS. The rod could pivot on the vertical plane to be operated, enabling the participant to raise and lower the foldable roll-bar. The rod was positioned at an overall height of 2425 mm from the ground, and it had a length of 800 mm. The end of the rod was covered by rubber to make gripping it easier (handgrip of about 115 mm). The gas spring (AMA, Reggio Emilia, Italy) used in the study had a stroke of 134 mm, a maximum arm length of 350 mm and a force of 350 N. In the present study, the rod was placed on the right side of the FROPS, and the gas spring was on the left (Figure 3). The mass of the upper inverted U-shaped folding steel tube of the FROPS was 22,550 kg and 27,450 kg for the conventional FROPS and for the prototype fitted with the newly designed handling system, respectively.

### 2.3. Procedure and Instruments

The trials took place at University of Torino’s Department of Agriculture, where the two mock-ups were placed at about 4 meters from each other on a straight surface. The participants were required to perform a total of four tasks:

1. Lowering the conventional FROPS (Task 1): the participants had to lower the FROPS while standing on the mock-up tractor near the operator’s seat;
2. Raising the conventional FROPS (Task 2): the participants had to raise the FROPS while standing on the back of the mock-up tractor;
3. Lower the enhanced FROPS (Task 3): the participants had to lower the FROPS while standing on the back of the mock-up tractor using the handling system;
4. Raise the enhanced FROPS (Task 4): the participants had to raise the FROPS while standing on the ground at the back of the tractor using the handling system.

The participant’s position to operate the FROPS was selected based on results from previous studies, since the mock-ups referred to a standard tractor [5] where no standardized ergonomic requirements exist [39], unlike for narrow-track tractors [3,4]. In particular, the conventional FROPS was lowered (Task 1) while standing near the operator’s seat, following the behavior observed by Caffaro et al. [14] and Micheletti Cremasco et al. [23], while the enhanced FROPS was lowered (Task 3) while standing on the back of the tractor based on the spontaneous behavioral strategies adopted by farmers to overcome reachability issues, as observed by Vigoroso et al. [24]. For the FROPS-raising operation (Task 2 and Task 4), the participants could keep their feet on the ground or use some parts of the tractor as a support for the feet, based on the behaviors observed by Micheletti Cremasco et al. [23] in their field study. In this latter case, they were instructed to maintain a three-point contact with the machine while handling the FROPS, in accordance with the health and safety regulations [40]. Furthermore, to ensure the participants’ safety, aside from providing a larger flat surface for the feet compared with the one usually available on a real tractor (see [24]), they were also observed by a technician who was ready to intervene in case any problems arose. To account for possible habituation or learning effects, half of the participants were randomly selected to operate the conventional FROPS first, while the remaining half operated the enhanced FROPS first. The participants conducted the tasks individually without having observed or been observed by the other participants.

Different instruments were used to evaluate the usability of the two FROPS:

1. An ad hoc questionnaire was developed starting from the NASA Task Load Index (TLX) [41] and adjusted for the targeted tasks. Originally developed by Sandra Hart of the NASA Ames Research Center (ARC) in the 1980s, the NASA TLX has become the gold standard for measuring subjective workload across a wide range of applications, and it has been validated in different fields of knowledge [42]. Adopting a multidimensional assessment procedure, the NASA TLX derives an overall workload score based on a weighted average of the ratings across six subscales: mental demand, physical demand, temporal demand, performance, effort and frustration. In the questionnaire developed for the present investigation, the participants were asked to rate their agreement (on a 4-point scale, from 1 (do not at all agree) to 4 (totally agree)) with different statements regarding the perceived usability of FROPS operation: perceived effort (Q1, Q2 and Q3), comfort and safety of the adopted posture (Q4 and Q5), satisfaction with the performance (Q6, Q7 and Q8) and perceived time demand (T1). The questions were designed to represent those factors detected as relevant in affecting the correct FROPS operation in previous studies [14,23], and they were proposed after every single task had been completed (see Table 1).
2. Observation of the operators’ behavior when raising and lowering the FROPS was performed. Observations were video-recorded using two orthogonal cameras stabilized on tripods: one placed on the right side of the mock-ups (side view) and the second one behind it (back view). The observations allowed for the detection of data concerning effectiveness (defined as the “accuracy and completeness with which users achieve specified goals” ([30], p. 2)) and efficiency (defined as the “resources expended in relation to the accuracy and completeness with which users achieve goals” ([30], p. 2)) in the interaction with the FROPS. A sociodemographic form investigating the participants’ ages, statures and body masses closed the questionnaire. No personal data dealing with Data Regulation (EU) 2016/679 were collected or processed.

### 2.4. Data Analysis

The mean ratings given by the participants for each item of the questionnaire were computed. A series of exploratory factor analyses (EFAs) with a Varimax rotation was performed on the scores obtained by items Q1–Q8 for both the conventional FROPS and the enhanced FROPS in both the raising and lowering tasks. Factor analysis is a technique used to identify groups or clusters of variables, and in this case, it was used to reduce a data set to a more manageable size while retaining as much of the original information as possible. Cronbach’s *α*, which is a measure of internal consistency (i.e., how closely related a set of items is as a group), represents the most common measure of scale reliability [43]. Thus, Cronbach’s *α* was then computed to measure the reliability of the extracted factors. Cronbach’s *α* was expressed as

$$\alpha =\frac{N^{2}\bar{Cov}}{\sum s_{item}^{2}+\sum Cov_{item}}$$

Values above 0.6 were accepted, mainly due to the small number of items that contributed to the factors [44]. The extracted factors and the time demand item were then compared through a series of paired sample *t*-tests to investigate the possible differences between the conventional and the enhanced FROPS. Indeed, this test is usually used when there are two experimental conditions and the same participants take part in both conditions of the experiment [43]:

$$t=\frac{\bar{D}-\mu_{D}}{s_{D}/\;\sqrt{N}}$$

The analyses were performed using IBM SPSS Statistical Package for Social Science v. 26 (IBM Corp., Armonk, NY, USA). Regarding the observations, descriptive statistics for effectiveness and efficiency were computed. For the effectiveness evaluation, the percentage of participants able to complete each of the four tasks autonomously was recorded, whereas for the efficiency evaluation, we analyzed the following:

- Posture: the percentage of participants who adopted risky, awkward and unbalanced postures in operating the FROPS (e.g., forward bending or twisting of the trunk);
- Procedure: (1) the percentage of participants (a) climbing on parts of the tractor structure to perform the tasks, (b) changing their hand-grasping position on the roll-bar or feet placement and (c) accompanying the FROPS in its final position and (2) the number of attempts before being able to pull down or raise the foldable roll-bar.

## 3. Results

### 3.1. Perceived Usability

Descriptive statistics for the investigated items in both the lowering and raising tasks for the conventional and the enhanced FROPS are reported in Table 2, while Table 3 shows the factor loadings and the results of the reliability analysis. The EFA yielded three factors which showed good reliability for all four tasks and which can be labeled as “effort” (F1), “physical discomfort” (F2) and “satisfaction” (F3).

Table 4 shows the results of the paired sample *t*-test analysis for the extracted factors and for the time demand item. Overall, for both the raising and lowering operations, the prototyped handling system reported statistically significant positive results for each factor investigated; lower effort, less physical discomfort, a higher level of satisfaction and a lower time demand were perceived.

### 3.2. Observed Usability

#### 3.2.1. Effectiveness

As can be seen in Table 5, the raising and lowering of the conventional FROPS required the intervention of the technician assisting the tests in helping some participants accomplish the tasks. Instead, tasks performed using the enhanced FROPS were carried out in complete autonomy and carried out effectively by all the participants.

#### 3.2.2. Efficiency for the Lowering Tasks

**Figure 4:**
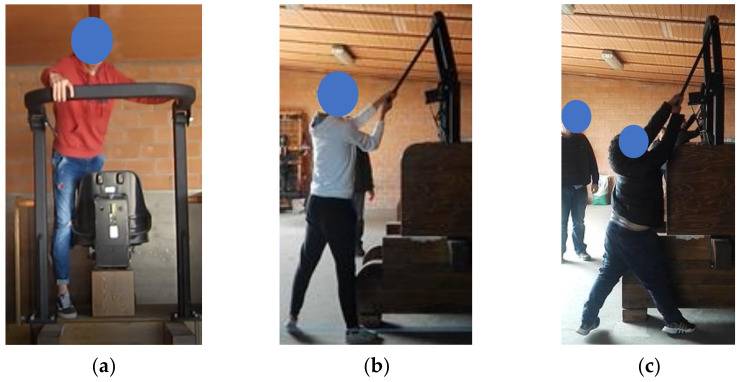
Participants when lowering (**a**) the conventional FROPS standing on the mock-up tractor near the operator’s seat (Task 1) and tall (**b**) and short (**c**) participants lowering the enhanced FROPS (Task 3) while standing on the ground at the rear right of the mock-up tractor.

To lower the conventional FROPS when standing near the seat on the driving station, the participants bent the trunk forward to support the mass of the FROPS, exposing themselves to an unbalanced posture (Figure 4a). Regarding the procedure to complete the task, nearly all the participants accompanied the roll-bar to its final position. Then, regarding the number of attempts performed to complete the required task, 6.5% of the participants changed the grasping position of their hands twice before lowering the FROPS. On the other hand, no critical safety issues were highlighted when using the rod to lower the enhanced FROPS (Figure 4b). Regarding the procedure to operate the enhanced FROPS, almost all the participants used two hands to grasp the rod, with some difficulties observed among the shorter participants (Figure 4c), but they were able to correctly and safely complete the task as well. Table 6 reports the main results.

#### 3.2.3. Efficiency for the Raising Task

**Figure 5:**
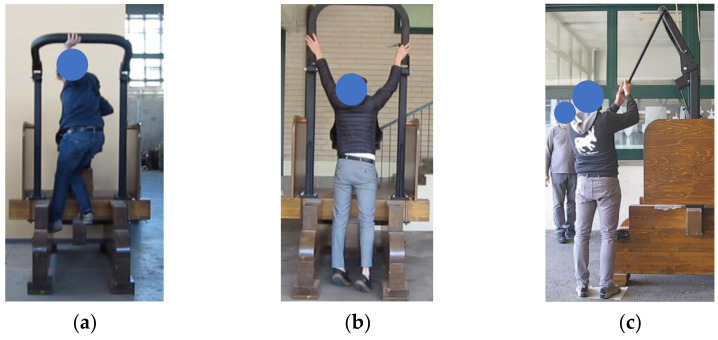
Participants when raising the conventional FROPS (Task 2) (**a**) adopting unbalanced postures standing on rear parts of the mock-up tractor structure, (**b**) having difficulty holding the FROPS firmly and (**c**) raising the enhanced FROPS (Task 4) from the ground by firmly grasping the rod with both hands.

When asked to raise the conventional FROPS, most of the participants climbed on parts of the simulated tractor or had difficulty holding the FROPS firmly (Figure 5a,b). Furthermore, three participants unsuccessfully tried to raise the FROPS while maintaining their feet on the ground and then completed the task by climbing on parts of the simulated tractor. When considering the number of attempts performed to complete the required task on the conventional FROPS, 10.7% of the participants made more than one attempt while changing their hands’ grasping position or their feet placement.

Regarding the raising task on the enhanced FROPS, the unsafe behaviors described in Section 2.2 did not occur, since the handling movement was carried out by all the participants with both feet on the ground, allowing them to firmly hold the rod connected to the FROPS (Figure 5c). Most participants identified the rod as the device to be used for lowering and raising the FROPS without requiring any explanation for its correct use. Five of them attempted some trials before understanding how to use it. Table 7 shows the main results.

## 4. Discussion

To promote a UCD approach to tractor safety, in the present study, we tested the usability of the prototype of a new handling system to facilitate the raising and lowering operations of a rear-mounted FROPS by comparing a conventional FROPS and a FROPS equipped with the newly designed device. Although the rod and gas spring design could be further improved, the solution is promising, and many positive considerations arose. First, all the UCD phases allowed for highlighting criticalities in the current FROPS handling methods, identifying the mechanisms which could be useful for developing the new design solution [14,24]. The proposed handling system could be considered a good design solution, since it has been proven to be intuitive and easy to use [45]. These considerations were mirrored by the observations and the average scores reported by the questionnaire. For both the raising and lowering tasks, the adoption of the rod and the gas spring allowed the participants to avoid awkward postures and perceive less physical discomfort, effort and time demand, which were retained as more satisfying. Furthermore, adopting such a solution moved the operators away from the pitching risks that may occur when manually moving the FROPS. All users involved in this trial experience could perform both the raising and lowering tasks more comfortably with the enhanced FROPS. Furthermore, during the raising task (that may be considered the most critical task in FROPS handling as a whole due to the mass and height of the foldable roll-bar), the participants showed less uncertainty in movements. Furthermore, fewer attempts were performed with the enhanced FROPS in comparison with the conventional one. These results are encouraging, and further investigations are needed to test the convenience of the proposed solution among professional farmers.

The presence of a simple rod pushed the participants to try it and use it successfully.

Based on a nudging perspective, a concept based on positive and indirect suggestions influencing the users’ behaviour and decision making [46], providing an alternative option which is not particularly expensive, leaving the operators the free will of choice and allowing them to perform the tasks more easily and safely could be a starting point to change operators’ default behaviors and attitudes toward FROPS handling. It has to be acknowledged that even though the expected improvement in the operation of the enhanced FROPS was not declared to the participants, simply seeing the two different FROPS may have raised some response biases, leading participants to answer in an affirmative manner. However, the validity of our results is supported by the triangulation and convergence of information from different sources—both the questionnaires and observations [47]—which support the increased usability of the enhanced system. Changes in the technical features or a new design should also be supported by targeted information and training activities to promote the correct behavior and use of the proposed solution. Promoting further information campaigns concerning FROPS adoption and training farmers through hands-on demonstrations of the correct and safe FROPS handling would be relevant. Indeed, more engaging training methods allow greater knowledge acquisition and more training transfer to the work setting, thereby improving behavioral safety performance and reducing negative safety and health outcomes [48].

However, concerning the reachability issue, even though it was significantly improved during the raising task, especially for shorter users, it was noticed that some further improvements should be made for the lowering task. For instance, the rod length should be increased to allow better performance among users with shorter statures if they decide to lower the FROPS while standing at the back of the tractor. Following this last consideration, a limitation of the present study could be that the lowering task for the enhanced FROPS was performed only while standing at the back of the tractor, whereas for the conventional FROPS, this was only performed from the tractor seat. However, the reasons lie in the results of the early stages of the UCD approach followed in this project [14,23,24].

The present study was performed on a tractor mock-up, and it was addressed toward evaluating only the system to facilitate the raising and lowering operations of a rear-mounted FROPS. In case such a solution should be adopted for a FROPS intended to be placed on the market, it shall be engineered to meet the safety requirements and general provisions of the current ROPS mandatory testing procedures. In case the devices should be installed on an in-use FROPS, the components have to be connected to the FROPS body without drilling or welding and firmly placed outside the clearance zone to avoid discrediting the results of the ROPS tests performed on the original FROPS and compromising the protection of the operator in case of overturning.

In addition, considering that the agricultural workforce is increasingly composed of operators who have different biomechanical, dimensional and functional characteristics (i.e., the elderly, migrants and women [49,50,51,52]), it is strongly recommended to consider the existing high degree of variability among the workforce population in the design of the handling tool. However, bearing in mind that it is not possible to design machinery that will suit every single individual, the adjustments for the prototype could be made while designing for the 5th–95th percentiles of the anthropometric spectrum [53], considering the body measurements of both male and female users.

Finally, the present research investigated the usability of the different FROPSs in a group of male users who may be considered as novices, based on Kumar et al. [54], who set a minimum of 5 years of tractor driving experience to be considered as an expert driver, and on Ferrari and Cavallo [55], who defined as novice users those who operated tractors only a few times a year. Future development of the research could further investigate the role played by previous experience in the perceived usability of FROPS operations by comparing novice and expert users [56]. In addition, gender differences could be assessed by involving a group of female drivers. An additional limitation should be acknowledged. The focus of the present study was to test the usability of a prototype of a new system to improve the comfort in use and to avoid health and safety risks during the raising and lowering operations of a rear-mounted FROPS. Even though the mock-up tractors and FROPS were equipped with a pin locking system, it was only used to keep the FROPS firmly in the upright position. However, since pin manual locking and retaining represent part of the FROPS operation process, to have a more complete list of criticalities while interacting with the device, the insertion and exclusion of the pins could be added as an additional task and evaluated in future studies.

## 5. Conclusions

The involvement of users in the early phase of the design and the evaluation of a prototype has proven to be useful for developing a handling system which makes rear-mounted FROPS operation more usable and may encourage its correct and safe use. Moreover, considering the anthropometrics differences existing in the farming population (in terms of gender, stature, strength and joint mobility), it is relevant to design machinery and equipment for as broad a range of users as possible to increase safety for as many operators as possible.

## Figures and Tables

**Table 1:** Questions used to evaluate the usability during the FROPS interaction.

ID	Items	Response
Q1	It was strenuous	4-point scale (1 = do not at all agree; 4 = totally agree)
Q2	It required a lot of physical effort	
Q3	The FROPS was heavy	
Q4	It was difficult to find support for my feet	
Q5	It was difficult to find support for my hands	
Q6	I immediately knew how to do it	
Q7	I manage the task in full autonomy	
Q8	I am satisfied with how I accomplished the task	
T1	It was time-demanding	

**Table 2:** Mean scores for all items used in the questionnaire.

Tasks	Items								
	Q1	Q2	Q3	Q4	Q5	Q6	Q7	Q8	T1
Task 1	2.05	1.75	2.16	1.34	1.41	3.40	3.15	3.34	1.76
Task 2	1.54	1.43	1.63	1.18	1.14	3.62	3.39	3.57	1.17
Task 3	1.97	1.85	2.14	1.45	1.43	3.43	3.23	3.28	1.73
Task 4	1.60	1.46	1.62	1.10	1.12	3.60	3.30	3.56	1.26

**Table 3:** Exploratory factor analysis for each task (Q1–Q8).

	Task 1			Task 2			Task 3			Task 4		
Item	F1	F2	F3	F1	F2	F3	F1	F2	F3	F1	F2	F3
Q1	0.810			0.835			0.833			0.744		
Q2	0.867			0.798			0.877			0.865		
Q3	0.754			0.882			0.862			0.841		
Q4		0.923			0.910			0.857			0.925	
Q5		0.918			0.901			0.844			0.923	
Q6			0.722			0.665			0.766			0.620
Q7			0.755			0.883			0.873			0.867
Q8			0.773			0.705			0.837			0.780
Explained variance (%)	34.071	14.685	21.342	28.519	24.804	23.019	29.051	19.551	25.973	26.598	23.964	23.030
Cronbach’s alpha	0.768	0.867	0.623	0.833	0.882	0.680	0.825	0.660	0.768	0.740	0.900	0.669

**Table 4:** Results for paired sample *t*-test for the three factors detected and the time demand item.

Task	Factors	*t*-Test
Effort	Task 1 vs. Task 3	6.123 ***
	Task 2 vs. Task 4	4.461 ***
Physical discomfort	Task 1 vs. Task 3	2.895 **
	Task 2 vs. Task 4	5.005 ***
Satisfaction	Task 1 vs. Task 3	−3.318 ***
	Task 2 vs. Task 4	−2.377 *
Time demand	Task 1 vs. Task 3	6.098 ***
	Task 2 vs. Task 4	5.042 ***

Note. * *p* < 0.05. ** *p* < 0.01. *** *p* < 0.001.

**Table 5:** Effectiveness evaluation for the four tasks considered.

Task 1 vs. Task 3		Task 2 vs. Task 4	
11% of participants were helped to support the FROPS	100% of participants carried out the task in complete autonomy	1 participant did not complete the task; 2 participants were helped by technician	100% of participants carried out the task in complete autonomy

**Table 6:** Efficiency evaluation for the lowering task for the two FROPS.

	Task 1	Task 3
Posture	97.8% * of the participants performed a forward bending of the trunk	No forward bending of the trunk
Procedure	6.5% of participants changed twice their hands’ grasping position, and 97.8% * of participants accompanied the roll-bar to its final position	100% of participants maintained their feet on the ground and accompanied the roll-bar to its final position, 64.5% of participants lowered the FROPS on the first or the second attempt, and 28% performed from 3 to 5 attempts and 7.5% from 6 to 10 attempts

* Two participants just pushed the foldable roll-bar and let it fall down.

**Table 7:** Efficiency evaluation for the raising task for the two FROPS.

	Task 2	Task 4
Posture	67.7% of the participants bent their trunk	No trunk bending or twisting
Procedure	67.7% of participants climbed on parts of the simulated tractor, 5% of participants maintained one foot on the ground and the other one on the 3-point lower links or rear power take-off protection or had difficulty holding FROPS firmly, 3 participants unsuccessfully tried to raise the FROPS while maintaining their feet on the ground and then completed the task by climbing on parts of the simulated tractor, and 10.7% of participants made more than one attempt	All the participants kept both feet on the ground and held the FROPS firmly, 79.6% of the participants raised the FROPS by gripping the rod with both hands, 19.3% used one hand only, and 5.3% of participants tried twice to find the best way to use the rod

## Data Availability

Data available on request due to privacy restrictions. The data presented in this study are available on request from the corresponding author. The data are not publicly available to protect participants’ privacy.
